# Inhibition of PTP1B Promotes M2 Polarization via MicroRNA-26a/MKP1 Signaling Pathway in Murine Macrophages

**DOI:** 10.3389/fimmu.2019.01930

**Published:** 2019-08-14

**Authors:** Xiaolong Xu, Xuerui Wang, Yuhong Guo, Yunjing Bai, Shasha He, Ning Wang, Yan Lin, Marc Fisher, Qingquan Liu, Yongming Yao

**Affiliations:** ^1^Trauma Research Center, Fourth Medical Center of the Chinese PLA General Hospital, Beijing, China; ^2^Department of Pathology, First Hospital Affiliated to the Chinese PLA General Hospital, Beijing, China; ^3^Beijing Hospital of Traditional Chinese Medicine, Capital Medical University, Beijing Institute of Traditional Chinese Medicine, Beijing, China; ^4^Beijing Key Laboratory of Basic Research With Traditional Chinese Medicine on Infectious Diseases, Beijing, China; ^5^The Department of Neurology, Beth Israel Deaconess Medical Center, Harvard Medical School, Boston, MA, United States

**Keywords:** sepsis, PTP1B, MKP1, miR-26a, murine macrophage, polarization

## Abstract

Sepsis is a life-threatening condition that often occurs in the intensive care unit. The excessive activation of the host's immune system at early stages contributes to multiple organ damage. Mitogen-activated protein kinase phosphatase-1 (MKP1) exerts an important effect on the inflammatory process. In our recent bioinformatic analysis, we confirmed that the inhibition of protein tyrosine phosphatase-1B (PTP1B) significantly promoted the expression of MKP1 in murine macrophages. However, the underlying mechanism and its effect on macrophage polarization remain unclear. In this study, we show that the suppression of PTP1B induced upregulation of MKP1 in M1 macrophages. A RayBiotech mouse inflammation antibody assay further revealed that MKP1-knockdown promoted pro-inflammatory cytokine (IL-1β, IL12p70, IL-17, IL-21, IL-23, and TNF-α) secretion but suppressed anti-proinflammatory cytokine (IL-10) production in M2 macrophages. Phospho-proteomics analysis further identified ERK1/2 and p38 as downstream molecules of MKP1. Moreover, we found that the inhibition of PTP1B lowered the expression of miR-26a, showing a negative correlation with MKP1 protein expression. Thus, we concluded that the inhibition of PTP1B contributes to M2 macrophage polarization via reducing mir-26a and afterwards enhancing MKP1 expression in murine macrophages.

## Introduction

Sepsis is a life-threatening condition seen in intensive care units (ICU) that is caused by a disordered reaction to infection (1, 2). Despite the development of supportive therapies, early treatment remains insufficient. The overall mortality of sepsis found in a recent cohort investigation involving 8,568 septic patients in the United States, was 15.7% (3). Although a new definition of Sepsis-3.0 diminishes the significance of the systemic inflammatory response syndrome (SIRS), the mainstream view suggests that sepsis is caused by inappropriate regulation of the immune system (1). A severe inflammatory response early after onset is essential for the excessive infiltration of inflammatory cells, leading to damage of tissue structure and dysfunction of organs (4).

Macrophages are critically involved in the innate immune response of the host via recognizing and presenting antigens and releasing cytokines as well as in the activation and resolution of inflammation (5, 6). Based on their plasticity, macrophages are divided into two classical phenotypes. M1 macrophages, activated by lipopolysaccharide (LPS) or interferon (IFN)-γ, promote the pro-inflammatory response by producing cytokines and inflammatory factors. Alternatively, M2 macrophages, stimulated by interleukin (IL)-4 or IL-10, are involved in the response to inflammation and wound healing (7). Recent studies indicated that the modulation of macrophage phenotype might be an appropriate strategy for treatment of sepsis (8, 9).

Mitogen-activated protein kinase phosphatase-1 (MKP1), encoded by the *Dusp1* gene, is also known as dual-specificity phosphatase-1 (DUSP 1). It belongs to the family of dual-specificity phosphatases and is important to the dephosphorylation of MAPKs, including p38 and JNK (10, 11). Due to the key role of MAPKs in the activation of inflammation, MKP1 is essential to the resolution of inflammation (12, 13). Our recent investigation found that the inhibition of protein tyrosine phosphatase-1B (PTP1B, encoded by *Ptpn1* gene) by punicalagin (PUN) promoted M2 macrophage polarization via suppression of the activation of MAPK family members and promotion of the anti-oxidative capacity (14). Moreover, we observed upregulated gene expression of MKP1 in both the PTP1B knockdown and punicalagin (PUN)-treated macrophage cell lines. Therefore, understanding the roles of MKP1 in macrophage activation and inflammation is critically important to the clarification of the mechanisms of MKP1 expression in the absence of PTP1B.

In this study, we established that the inhibition of PTP1B induced the expression of MKP1 and promoted a tilt toward the M2 phenotype, which was partially reversed in the *Dusp1*-knockdown cell line. ERK1/2 and p38 were involved in the functional switch of macrophages. Furthermore, downregulation of miR-26a was observed in the absence of PTP1B, which increased MKP1 expression. Our findings suggest that the prevention of the activity of PTP1B decreases the production of miR-26a, which in turn enhances the expression of MKP1 and promotes M2 macrophage polarization. Our present findings provide novel evidence further elucidating the role of the PTP1B/MKP1 signaling pathway in macrophage-involved diseases.

## Materials and Methods

### Reagents

LPS (*Escherichia coli* O55:B5), LY3214996, AX15836, and p38 IN-1 were purchased from MedChemExpress LLC (Shanghai, China). Punicalagin [>98% high-performance liquid chromatography (HPLC) purity] was purchased from Tauto Biotech (Shanghai, China). Fetal bovine serum (FBS), Dulbecco's modified Eagle medium (DMEM), and antibiotic-antimycotic were obtained from Gibco (Grand Island, NY, USA). The ELISA kits for IL-1β and TNF-α were bought from Cusabio (Wuhan, China). Bicinchoninic acid (BCA) protein assay kit was purchased from Pierce (Rockford, IL, USA). Antibodies for GAPDH, PTP1B, MKP1, p-ERK1/2, p-ERK5, p-p38, iNOS, and Arg-1 were produced by Cell Signaling Technology (Danvers, MA, USA). The goat anti-mouse antibody was purchased from Li-cdr Odyssye® (Lincoln, NE, USA). The assay kit used was manufactured by RayBiotech, Inc. (Norcross, GA, USA).

### Animals

Male C57BL/6J mice were purchased from the Nanjing Biomedical Research Institute of Nanjing University. All experimental procedures were approved by the Animal Care and Use Committee of Beijing Hospital of Traditional Chinese Medicine, and efforts were made to minimize the discomfort of the animals.

### Knockout of *Ptpn1* Gene in Mice by CRISPR/Cas9 System

The *Ptpn1*-knockout (KO) mice were generated in the Nanjing Biomedical Research Institute of Nanjing University (Nanjing, China). The transcript of Ptpn1-201 was selected for the generation of KO animals, which had a C57BL/6J background. *Ptpn1*-KO mice were generated by using a clustered regularly interspaced short palindromic repeats (CRISPR)/CRISPR-associated (Cas)9 system. Guide (g)RNAs direct Cas9 endonuclease cleavage of the Ptpn1 gene and create a double-strand break. Deletion of exon 2-8 of the *Ptpn1* gene using the CRISPR/Cas9 system induces a frameshift mutation. Four suitable gRNAs have been designed in intron1-2 and intron8-9 of Ptpn1-201: S1:TGAACAGAAGCACCACATAGG;S2:ACAGCCCAGCAAGAACAGGG; S3:CCTGCAGGAAGTGGCACTGTGG;S4:TCATTTCACAGGAACATCCA. Additionally, guide (g)RNAs were constructed *in vitro*. Then Cas9 mRNA and sgRNA were co-injected into zygotes. Thereafter, the zygotes were transferred into the oviduct of pseudopregnant ICR females at 0.5 dpc (day post-coitum). F0 mice were born after 19~21 days of transplantation. All the offspring of ICR females (F0 mice) were identified by PCR and sequencing of tail DNA. Positive F0 mice which had a copy of the deletion exon2-8 of *Ptpn1* gene were genetyped. Two lines of mice, documented as *Ptpn1*-12725bp and *Ptpn1*-12735bp, were employed in this study. Finally, crossing F0 mice with C57BL/6J mice were performed to produce heterozygous mice.

### Cell Culture

A RAW264.7 macrophage cell line was purchased from the American Type Culture Collection (Rockville, MD, USA) and cultured in DMEM medium supplemented with 10% FBS and antibiotics (100 U/mL of streptomycin and 100 U/mL of penicillin) at 37 °C in a humidified incubator with 5% CO_2_. Mice peritoneal macrophage was collected as described previously (14). Briefly, thioglycolate broth (4%) was intraperitoneally injected 3 days before operation. The mice of each treatment group were quickly sacrificed and peritoneal cells were harvested after intraperitoneal injection of cold PBS. Cells were then incubated on dishes (2 × 10^6^ cells/dish) for relative experiments.

### Establishment of Cecal Ligation Puncture (CLP) Mice Model

Eighty male C57BL/6J mice 8 weeks old (18–22 g) were randomly divided into four treatment groups. CLP surgery was performed following a protocol as described before (15). Briefly, mice were fasted for 24 h with water permitted before surgery. Mice were anesthetized and the abdominal cavity was opened. A 5-gauge needle was used to puncture in the cecum (1 cm from ileocecal junction) one time. Next, the cecum was cautiously relocated into the abdomen and the incision was sewed up. Finally, the mice were injected with 1 mL 37°C normal saline subcutaneously and fasted for another 12 h.

### Knockdown of *Ptpn1* and *Dusp1* in RAW264.7 Cells

We used a pGMLV-SC5 RNAi system to knockdown the gene expression of *Ptpn1* (14) and *Dusp1*. Briefly, oligonucleotides encoding the shRNAs for *Ptpn1* and *Dusp1* were annealed and ligated into the EcoRI and BamH sites of pGMLV-SC5 vectors. Then, the RNAi plasmids were transfected into 293T cells to generate a lentivirus. Eventually, the lentivirus carrying M_*Ptpn1*-shRNAs, *Ptpn1*-Vector-NC, M_*Dusp1*-shRNAs, and *Dusp1*-Vector-NC were collected and inserted into RAW264.7 cells. The protein expression levels of PTP1B and MKP1 were examined 24 h later by Western blot. For MKP1, three *Dusp1* shRNAs were designed as listed. (*Dusp1* shRNA-1: GATCAACGTCTCAGCCAATTG; *Dusp1* shRNA-2: GCT CCACTCAAGTCTTC TTTC; *Dusp1* shRNA-3: GCTTACCTCATGAGGACT AAC).

### Microarray-Based Transcriptional Profiling Analysis

RNA sequencing was performed using an Illumina HiSeq™ 2000 system. The RNA samples obtained from each group were first treated by DNase I to degrade any possible DNA contaminants and to release oligonucleotides. Then, the oligonucleotides were enriched by oligo (dT) and fragmented into short fragments. Afterwards, we qualified and quantified the sample library, which had been prepared for sequencing via Illumina HiSeq™ 2000, using an Agilent 2100 Bioanalyzer and ABI Real-Time PCR. Further analyses based on DEGs were employed to discover the gene changes at the transcriptional level.

### Cytokine Microarray Analysis

Supernatants of each treatment group were assayed using a RayBio® mouse inflammation antibody array (RayBiotech AAM-INF-1, Norcross GA, USA). Briefly, supernatants were diluted at two factors, followed by overnight incubation in antibody array pools. The array glass slides were washed twice and incubated in a biotin-conjugated anti-cytokine mix for 2 h. Further, Cy3-conjugated streptavidin was added into the antibody array pools, followed by incubation for another 2 h. Eventually, the intensity of fluorescence signals was assessed using a laser scanner (InnoScan 300 Microarray Scanner, Innopsys, Carbonne, France). The signal values were analyzed by a RayBiotech analysis tool.

### Phosphoproteomics Analysis

RAW264.7 cells were treated with the desired reagents and then lysed in a lysis buffer with a phosphatase inhibitor. The protein concentration was then measured using a BCA assay. Equal amounts of 20 ug of proteins of were separately collected from each treatment group and concentrated by SpeedVac for phosphopeptide enrichment. Titanium dioxide beads (TiO2) were employed for the enrichment of phosphopeptides as described before (16). Briefly, TiO_2_ beads were incubated with samples for 1 h at room temperature and then pelleted by centrifugation. After washing, these phosphopeptides were eluted and concentrated in SpeedVac. All samples were desalted by C18 and loaded into a Nano-LC system. Peptides were separated and MS/MS was performed in the normal mode using data-dependent acquisition. MS raw phosphoproteome data were analyzed by MaxQuant software. ANOVA testing was conducted to identify proteins that were significantly changed at the phosphorylation level.

### Enzyme-Linked Immunosorbent Assay (ELISA)

To examine the cytokine secretion, including that of IL-1β and IL-10, RAW264.7 macrophages were pre-incubated on 24-well plates (4 × 10^5^) for 12 h and then treated with the desired agents. The supernatants of each treatment group were collected, and detection for cytokines was immediately conducted using Cusabio ELISA kits. All protocols were performed as per the manufacturer's instructions.

### Western Blotting

RAW264.7 macrophages (1 × 10^6^) were pre-incubated for 24 h and treated with the desired agents. After trypsinization, the proteins were harvested from each treatment group using a lysis buffer and quantified via BCA assay. Proteins were initially separated by SDS-PAGE and electro-transferred to nitrocellulose membranes (Pierce, USA) to detect their expression level. Next, they were incubated with specific antibodies. The densitometric values were calculated based on three parallel tests using ImageJ software.

### Statistical Analysis

Data are expressed as mean ± S.E.M. The differences between the mean values of the normally distributed data were assessed by one-way ANOVA comparisons. A *P*-value of 0.05 or less was considered statistically significant.

## Results

### Inhibition of PTP1B Promotes MKP1 Expression

**Figure 1:**
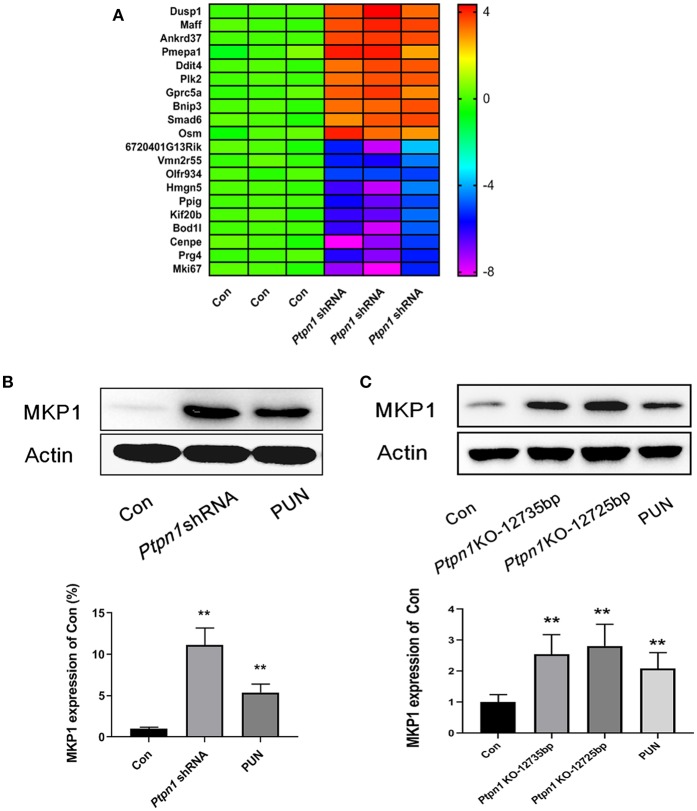
Inhibition of PTP1B leads to upregulation of MKP1. **(A)** Assessment of the downstream genes of PTP1B by transcriptional profiling. *Ptpn1* WT cells (Con) and knockdown cells were incubated for 24 h and harvested for transcriptional profiling analysis. Three independent experiments were performed and the fold changes (in log2 value) in the control group were found, which are presented in the heatmap. **(B)** Detection of the MKP1 expression in RAW264.7 cells. The cells of the control and *Ptpn1* knockdown groups were incubated for 24 h without specific treatment. For the PUN treatment group, *Ptpn1* WT cells were incubated with 20 μM PUN for 24 h. **(C)** Detection of the MKP1 expression in peritoneal macrophages. Peritoneal macrophages were obtained from indicated mice groups, including *Ptpn1* WT and *Ptpn1* knockout mice of two sequences. *Ptpn1* WT cells incubated with 20 μM PUN for 24 h were represented as PUN group. Data are expressed as mean ± SD of three independent experiments. **p < 0.01 indicates statistically significant difference compared with control group.

In our previous study, we characterized the role of PTP1B deficiency in promoting M2 macrophage polarization. However, the downstream molecules of PTP1B which switch the function of macrophages remain unclear. RNA sequencing analysis was employed to uncover the potential downstream genes associated with PTP1B deficiency in macrophages. After transcriptional profiling data analysis, the gene *Dusp1* attracted our attention with its highest amplification in the *Ptpn1* knockdown cells, compared with the *Ptpn1* wild-type (WT) cells (Fold change = 13.83) (Figure 1A). Subsequently, detection based on Western blot further confirmed the enhanced expression of MKP1 in RAW264.7 cells (Figure 1B). Similarly, the inhibition of PTP1B by the chemical inhibitor PUN (IC_50_ = 1.04 μM) also significantly promoted the expression of MKP1 at the translational level as compared with that of the control group (Figure 1B). To physiologically demonstrate the upregulation of MKP1 induced by PTP1B inhibition, peritoneal macrophages from *Ptpn1* WT and *Ptpn1* knockout mice (−12,735 bp and −12,725 bp) were employed to determine the expression of MKP1. The results showed that inhibition of PTP1B by PUN treatment and a Cas9/KO knockout strategy at both sequences significantly upregulated MKP1 expression in murine macrophages (Figure 1C).

### Deficiency of MKP1 Prevents M2 Macrophage Polarization

**Figure 2:**
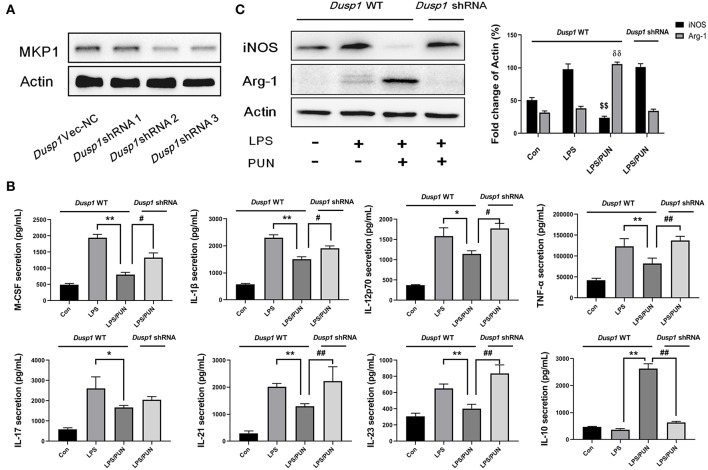
Deficiency of MKP1 prevents M2 macrophage polarization. **(A)** Identification of transfection efficiency of *Dusp1* shRNAs. The lentivirus carrying M_*Dusp1*-shRNAs and *Dusp1*-Vector-NC were designed and inserted into RAW264.7 cells. The protein expression levels of MKP1 were measured 24 h later by Western blot. **(B)** Detection of pro- and anti-inflammatory cytokines by RayBiotech assay. MKP1 WT and knockdown cells were pretreated with 20 μM PUN for 3 h, followed by a treatment with 1 μg /mL of LPS for 24 h, as indicated. The cytokine secretion was measured using a RayBiotech assay kit. Data are expressed as mean ± SD of three independent experiments. *p < 0.05, **p < 0.01, ^#^*p* < 0.05, and ^##^*p* < 0.01 indicate statistically significant differences between the groups; **(C)** MKP1 is involved in the expression of iNOS and Arg1. MKP1 WT and knockdown cells were pretreated with 20 μM PUN for 3 h and then treated with 1 μg/mL of LPS for 12 h, as indicated. Western blot was employed to assess the protein expression. Data are expressed as mean ± SD of three independent experiments. ^$$^*p* < 0.01 and ^δδ^*p* < 0.01 indicate statistically significant differences compared with the LPS group.

A stable *Dusp1* knockdown RAW264.7 cell line was established to verify our hypothesis that the induction of MKP1 is vital to M2 macrophage polarization. Three *Dusp1* shRNAs were designed and their transfection efficiency was detected by western blot. As shown in Figure 2A, transfection of vector and *Dusp1* shRNA1 had limited effects on the expression of MKP1 protein. Meanwhile, transfection of *Dusp1* shRNA2 and shRNA3 remarkedly decreased MKP1 expression in macrophages. Considering the efficiency of transfection and cell viability, *Dusp1* shRNA2 was selected for further studies. A RayBiotech protein array confirmed the presence of seven significantly downregulated proinflammatory cytokines in the PUN-treated M1 macrophages, including IL-1β, TNF-α, M-CSF, IL-12p70, IL-17, IL-21, and IL-23. Also, the secretion of the anti-inflammatory factor IL-10 was remarkably enhanced in PUN-stimulated M1 macrophages (Figure 2B). Consistent with these observations, data based on Western blot revealed that the PUN treatment prevented LPS-induced expression of the M1-polarizing marker iNOS and promoted the expression of the M2-polarizing marker Arg-1 (Figure 2C). However, the tendency toward M2 polarization was not observed in the *Dusp1*-knockdown RAW264.7 cell line. The reduced production of IL-1β, TNF-α, M-CSF, IL-21, and IL-23 by PUN treatment was reversed in *Dusp1*-knockdown macrophages (Figure 2B). PUN-induced expression of IL-10 was also suppressed due to MKP1 deficiency (Figure 2B). Moreover, the regulatory properties of PUN on the downregulation of iNOS and the upregulation of Arg-1 were restored in *Dusp1*-knockdown macrophages (Figure 2C).

### ERKs and p38 Are Negatively Regulated by MKP1 in Macrophages

**Figure 3:**
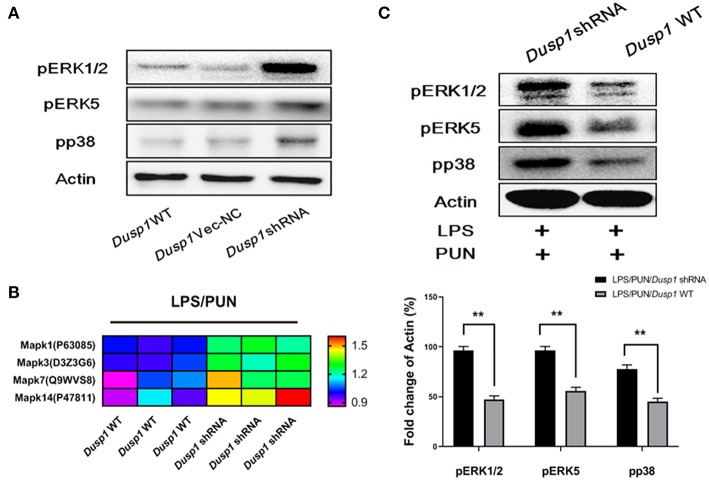
ERKs and p38 are negatively regulated by MKP1 in macrophages. **(A)** Effects of *Dusp1* interference on downstream MAPKs members. The lentivirus carrying M_*Dusp1*-shRNA and *Dusp1*-Vector-NC was designed and inserted into RAW264.7 cells. The phosphorylation levels of ERK1/2, ERK5, and p38 were measured 12 h later by Western blot. **(B)** Investigation on the phosphorylation level of downstream MAPKs members. *Dusp1* WT and knockdown cells were pretreated with 20 μM PUN for 3 h and then treated with 1 μg/ml LPS for 12 h, as indicated. The phosphorylation level of each MAPKs members was detected via phospho-proteomics approach. Three independent experiments were performed. The proteins with fold changes (in log2 value, of control group) higher than 1.2 are listed in the heatmap. **(C)** Identification of the phosphorylation of MAPKs members. *Dusp1* WT and knockdown cells were pretreated with 20 μM PUN for 3 h and then treated with 1 μg/mL of LPS for 12 h, as indicated. The phosphorylation level of each MAPK member was detected via Western blot. Data are expressed as mean ± SD of three independent experiments. **p < 0.01 indicates statistically significant differences between indicated groups.

Considering that MKP1 participates in the activation of MAPK family members by dephosphorylation modifications, we employed a quantitative phospho-proteomics approach to understand the role of MKP1 exerted in macrophage polarization. The phosphorylation level of MAPKs members in *Dusp1* WT, *Dusp1* Vector-NC, and *Dusp1* shRNA cells were detected by western blot. The data demonstrated that transfection of *Dusp1* Vector-NC has no significant effect on phosphorylation of downstream ERK1/2, ERK5, and p38, compared with *Dusp1* WT cells. However, deficiency of MKP1 promoted the phosphorylation of these proteins (Figure 3A). Data based on phospho-proteomics further showed that the treatment with PUN in *Dusp1*-knockdown macrophages significantly upregulated the phosphorylation level of MAPKs members, including Mapk1(ERK2), Mapk3(ERK1), Mapk7 (ERK5), and Mapk14 (p38), as compared with that in *Dusp1* WT cells (FC > 1.2) (Figure 3B). The results from the Western blot confirmed the enhanced phosphorylation level of ERK1/2, ERK5, and p38 in *Dusp1*-knockdown macrophages treated by PUN (Figure 3C).

### ERK1/2 and p38 Are Essential to MKP1-Mediated Macrophage Polarization

**Figure 4:**
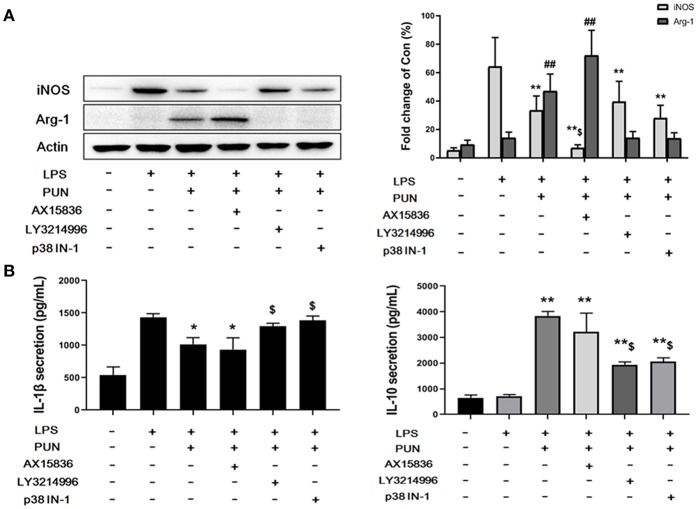
ERK1/2 and p38 are essential to MKP1-mediated macrophage polarization. **(A)** ERK1/2 and p38 are involved in the expression of iNOS and Arg-1. Cells were pretreated with 20 μM PUN with desired chemical inhibitors for 3 h and then treated with 1 μg/mL of LPS for 12 h, as indicated. The expression of iNOS and Arg-1 were detected by Western blot. Data are expressed as the mean ± SD of three independent experiments. **p < 0.01 and ^##^*p* < 0.01 indicate statistically significant differences compared with the LPS group. ^$^*p* < 0.05 indicates statistically significant differences compared with LPS/PUN group. **(B)** ERK1/2 and p38 are involved in the expression of IL-1β and IL-10. Cells were pretreated with 20 μM PUN with the desired chemical inhibitors for 3 h and then treated with 1 μg/mL of LPS for 24 h, as indicated. The expression of IL-1β and IL-10 were detected by ELISA. Data are expressed as mean ± SD of three independent experiments. *p < 0.05, **p < 0.01 indicate statistically significant differences compared with LPS group. ^$^*p* < 0.05 indicates statistically significant differences compared with LPS/PUN group.

To further confirm whether ERKs and p38, downstream factors of MKP1, participate in M2-polarizing induced by PTP1B suppression, cells were treated by 5 nM LY3214996 (inhibitor of ERK1 and ERK2), 8 nM AX15836 (inhibitor of ERK5), and 5 μM p38 IN-1 (inhibitor of p38), respectively. The results obtained show that treatment by LY3214996 and p38 IN-1 notably inhibited the production of M1-polarizing markers IL-1β and iNOS, and promoted generation of M2 markers IL-10 and Arg-1 in PUN-stimulated *Dusp1*-knockdown cells, compared with *Dusp1* WT group (Figures 4A,B). However, the inhibition of ERK5 by AX15836 exerted a limited regulatory effect on the phenotype switch.

### The Inhibition of PTP1B Promotes MKP1 via Decreasing miR-26a Production

**Figure 5:**
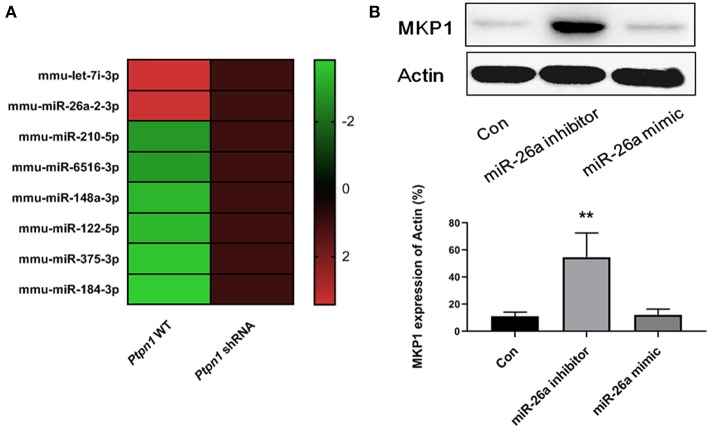
Inhibition of PTP1B promotes MKP1 via decreasing miR-26a production. **(A)** miRNAs expression profiles analysis. *Ptpn1* WT cells and knockdown cells were incubated for 24 h and then harvested for deep-sequencing analysis. Three independent experiments were performed and fold changes (in log2 value) of control group are listed in heatmap. **(B)** The expression of MKP1 is regulated by miR-26a. Cells were treated by miR-26a mimic or miR-26a inhibitor for 24 h respectively, and harvested for Western blot detection. Data are expressed as mean ± SD of three independent experiments. **p < 0.01 indicates statistically significant differences as compared with the control group.

Since miRNAs have been identified as essential factors in various biological processes, we conducted deep sequencing to find clues in the PTP1B-MKP1 signaling axis. We performed miRNAs expression profiles analysis and discovered that expression of miR-26a in the absence of PTP1B was significantly lower than that of the control group (Figure 5A). Additionally, a prediction based on the TargetScan 6.2 database provided evidence that MKP1 was a potential target of miR-26a. To establish whether miR-26a regulates MKP1 expression, we detected the levels of MKP1 protein in macrophages in the presence of a miR-26a mimic and in the presence of a miR-26a inhibitor, respectively. As expected, treatment with the miR-26a inhibitor promoted the expression of MKP1, but no increase was observed in control and miR-26a mimic groups (Figure 5B).

### PUN Treatment Prevents Sepsis-Induced Excessive Inflammation in Mice

**Figure 6:**
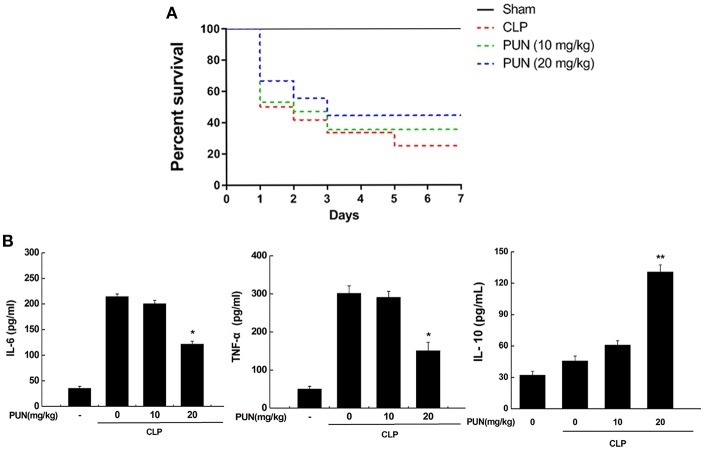
PUN treatment prevents sepsis-induced excessive inflammation in mice. **(A)** Kaplan–Meier curves for the survival rate of septic mice. Eighty mice were involved in the comparison. The dosages of PUN used in this study were indicated as 0, 10, and 20 mg/kg·bw, respectively. PUN was orally administrated at the indicated dosage 3 days ahead of CLP surgery. Then all mice had surgery with or without cecal ligation and puncture. The observation for mortality continued for 7 days. **(B)** ELISA detection on expression of serum cytokines (24 h after surgery). Data are expressed as mean ± SD of three independent experiments. *p < 0.05 indicates statistically significant differences as compared with the CLP surgery group. **p < 0.01 indicates statistically significant differences compared with LPS group.

To investigate if the PTP1B inhibitor has potential therapeutic properties in sepsis, we used CLP surgery to establish a septic mice model. Mice were pretreated with or without PUN (10, 20 mg/kg) for 3 days before CLP surgery. Survival data showed that PUN (20 mg/kg) treatment has promising effect on septic mice by significantly reducing the death rate throughout the 7 days of observation (Figure 6A). Results based on ELISA detection further illustrated that PUN treatment (20 mg/kg) notably decreased the secretion of IL-6 and TNF-α in the serum of septic mice at 24 h after surgery. Moreover, the production of IL-10 was increased by PUN treatment in septic mice at 24 h after surgery (Figure 6B).

## Discussion

Sepsis is an immunological disorder which causes multi-organ failure to patients and is a significant burden for healthcare system (17, 18). This life-threatening condition starts with an infection and at onset activates a systemic inflammatory response by generation of pro-inflammatory cytokines. The excessive inflammatory response induced by cytokines causes an initial hyperinflammatory “cytokine storm” and subsequently contributes to organ damage. In the advanced stage of the disorder, the protracted immunosuppressive phase due to unresolved septic foci causes, the majority of septic deaths are caused by compensatory secretion of anti-inflammatory cytokines (19, 20). Considering the damage caused by uncontrolled inflammation of the host immune system, intervention in the early stage of the inflammatory process may facilitate the prevention of sepsis development (21). Macrophage polarization is one of the host mechanisms for control of the proper direction of immune function (22, 23). It has the duality of a M1 phenotype for pro- and M2 phenotype for anti-inflammatory cytokines. Previously documented as a key regulator in diabetes, insulin resistance, and obesity (24–26), PTP1B has drawn increasing attention because of its important role in inflammatory diseases (25, 27). However, the issue of promoting or inhabiting effects of PTP1B on inflammation remains disputed. Xu et al. and Nasimian et al. have reported that inhibition of PTP1B results in increased phosphorylation of JNK, ERK, p38, and then enhancement of the inflammatory response in murine macrophages (28, 29). In contrast, Pardo et al. and Tsunekawa et al. demonstrated that reduced expression of PTP1B is associated with an attenuated inflammatory response in macrophages and microglia (27, 30). Although the underlying mechanism of PTP1B in inflammation remains unclear, several pharmacological studies suggest that inhibition of PTP1B by pharmacological intervention results in attenuated inflammation and a better outcome of pathological processes. Quang et al. demonstrated that SF-6013 suppresses the activity of PTP1B and thus inhibits activation of RAW264.7 cells (31). Daveri et al. reported that cyanidin and delphinidin inhibit inflammation via preventing the overexpression of PTP1B and activation of NF-κB (32). Our previous study also demonstrates that PUN treatment promoted M2 polarization via inhibition of the activity of PTP1B protein in macrophages (14). Drugs with PTP1B inhibitory property may have potential anti-inflammatory effects. Further studies are needed since different PTP1B inhibitors may have distinctly different regulatory mechanisms.

MKP1 is critically involved in the activation of macrophages (33, 34). However, our understanding of its specific roles in macrophage polarization is still extremely limited. Interestingly, we noticed significant upregulation of *Dusp1* at the transcriptional level in PTP1B-deficient cells via RNA sequencing analysis. Enhanced expression of MKP1 at translational level was also observed in the absence of PTP1B, verified by the PTP1B knockdown and inhibitor treatment. To further confirm this in a more physiological model, peritoneal macrophages collected from *Ptpn1* WT mice and *Ptpn1* knockout mice (−12,735 bp and −12,725 bp) were used for western blot detection. As expected, inhibition of PTP1B by PUN treatment and Cas9/KO technique contributes to the enhanced expression of MKP1 in murine peritoneal macrophages. These findings validated that the suppression of PTP1B leads to upregulation of MKP1 in macrophages. As mentioned earlier, the inhibition of PTP1B by PUN in macrophages induces a change toward the M2 phenotype, and thus our next aim was to confirm whether MKP1 participates in the switch of immunological function. We established a stable *Dusp1*-knockdown RAW264.7 cell line to facilitate our understanding of the role of MKP1 in macrophage polarization. The data of the RayBiotech protein assay revealed that the inhibition of PTP1B in the presence of LPS markedly decreased the production of the M1-polarizing marker iNOS and pro-inflammatory cytokines, including IL-1β, TNF-α, M-CSF, IL-12p70, IL-17, IL-21, and IL-23. Meanwhile, the generation of the M2 marker Arg-1 and anti-inflammatory IL-10 was increased, indicating a tendency toward the resolution of inflammation. However, this trend was repressed in the *Dusp1*-knockdown RAW264.7 cell line, induced by LPS. These results confirmed our hypothesis that MKP1 is an important regulator of the M2 phenotype switch, induced by the suppression of PTP1B.

ERK, JNK, and p38 are the three main arms of MAPKs regulating immune functional responses of macrophages to a wide array of stimuli (35–37). Given the crucial role of MKP1 in the dephosphorylating regulation of MAPKs, it has long been suspected that MKP1 may participate in the modulation of cytokine biosynthesis (38, 39). However, our knowledge of the effect of MKP1 in M2 polarization is still limited. Data from quantitative phospho-proteomics give us an unbiased comprehension of a variety of cascades of MAPKs. Our data showed that the phosphorylation level of ERK1, ERK2, ERK5, and p38 were enhanced in the absence of MKP1. Interestingly, JNK has been reported to be another vital downstream effector of MKP1 (40, 41), but our study shows that it is not involved in the M2 polarization, induced by PTP1B inhibition. The aforementioned inhibitors were employed to further reveal the effect of those identified regulators in the MKP1/MAPKs signaling pathway. LY3214996 and p38 IN-1, two inhibitors for ERK1/2 and p38 respectively, significantly repress PUN-induced M2 macrophage polarization in the presence of MKP1. However, the inhibition of ERK5 by AX15836 during the process of switching was limited. Taken together, based on the present findings, we conclude that promotion of MKP1 induced by the inhibition of PTP1B promotes the anti-inflammatory actions of macrophages via dephosphorylation of ERK1/2 and p38.

MicroRNA is a species of functional non-coding RNAs, which regulates targeting genes via reversibly binding to certain sequences. miR-26a is involved in numerous biological processes, including cancer, inflammation, and oxidative stress (42–44). However, scarce evidence exists about its biological activity on macrophage polarization. Our study established a decrease in the expression of miR-26a in the absence of PTP1B in macrophages, suggesting that miR-26a may be involved in macrophage polarization, which is mediated by PTP1B. However, miRNAs are commonly regulated at the transcriptomic level. Based on our understanding, it is likely that miRNA-26a is not a direct target of PTP1B in murine macrophages. We tried to understand the underlying mechanism of PTP1B/miR-26a by using certain approaches. Yet, no direct target of PTP1B that regulates the expression of miR-26a has been identified. By employing a prediction approach, we preliminarily deduced a relationship between miR-26a and MKP1. Further detection by Western blot confirmed that MKP1 is a downstream target of miR-26a since the miR-26a inhibitor enhanced the expression of MKP1 in macrophages.

In our recent study, we demonstrated that heme oxygenase-1 (HO-1) is an important downstream effector molecule that participates in the promotion of M2 polarization (14). Therefore, we tried to understand whether MKP1 was also involved in the upregulation of HO-1. However, the results showed that MKP1 knockdown alone does not result in an enhanced expression of HO-1. Similar results were observed in miR-26a mimic and/or inhibitor treatment (Data not shown). Therefore, we conclude that there is no direct relationship between MKP1 and HO-1. It has been well-established that PTP1B dephosphorylates JAK2 and IRS1, and thus modulates the phosphorylation of STAT3 and AKT. In our previous study (14, 45), we found that inhibition of PTP1B promotes the expression of HO-1 via STAT3- and AKT-mediated pathways. In a word, the promotion of HO-1 and of MKP-1 by PUN may be two separate pathways in the suppression of inflammation.

In conclusion, our investigation revealed that the inhibition of PTP1B promoted M2 polarization via lowering the production of miR-26a, which in turn enhanced MKP1 expression in murine macrophages. ERK1/2 and p38, two downstream effectors of MKP1, participated in the process of phenotype switching. Our findings provide a novel insight into the role of MKP1 in macrophage polarization and inflammatory diseases.

## Data Availability

The raw data supporting the conclusions of this manuscript will be made available by the authors, without undue reservation, to any qualified researcher.

## Ethics Statement

This study was carried out in accordance with the recommendations of “Committee for the Care and Use of Experimental Animals at Beijing Institute of Traditional Chinese Medicine.” The protocol was approved by the “Beijing Institute of Traditional Chinese Medicine.”

## Author Contributions

XX: substantial contributions to the conception and design of the work, drafting the work, and revising it critically for important intellectual content. XW: substantial contributions to the design of the work, interpretation of data for the work. YG, SH, YB, and NW: acquisition, analysis, and interpretation of data for the work. YL: drafting the work and revising it critically for important intellectual content. MF: language polishing and adjustment of structure of manuscript. QL and YY: substantial contributions to the design of the work, final approval of the version to be published, agreement to be accountable for all aspects of the work in ensuring that questions related to the accuracy and integrity of any part of the work are appropriately investigated and resolved.

### Conflict of Interest Statement

The authors declare that the research was conducted in the absence of any commercial or financial relationships that could be construed as a potential conflict of interest.
